# Xeno-free pre-vascularized spheroids for therapeutic applications

**DOI:** 10.1038/s41598-017-18431-6

**Published:** 2018-01-10

**Authors:** E. Bauman, T. Feijão, D. T. O. Carvalho, P. L. Granja, C. C. Barrias

**Affiliations:** 1 Instituto de Inovação e Investigação em Saúde (i3S), Universidade do Porto, Porto, Portugal; 2 Instituto de Engenharia Biomédica (INEB), Universidade do Porto, Porto, Portugal; 3 Faculdade de Engenharia da Universidade do Porto (FEUP), Porto, Portugal; 4 Instituto de Ciências Biomédicas Abel Salazar (ICBAS), Universidade do Porto, Porto, Portugal

## Abstract

Spheroid culture has gained increasing popularity, arising as a promising tool for regenerative medicine applications. Importantly, spheroids may present advantages over single-cell suspensions in cell-based therapies (CT). Unfortunately, most growth media used for spheroid culture contain animal origin-components, such as fetal bovine serum (FBS). The presence of FBS compromises the safety of CT and presents economic and ethical constraints. SCC (supplement for cell culture) is a novel xeno-free (XF) industrial cell culture supplement, derived from well-controlled pooled human plasma and processed under good manufacturing practice rules. Here, we developed a XF SCC-based formulation for 2D-culture of outgrowth endothelial cells (OEC), and then used it for generating co-culture spheroids of OEC and mesenchymal stem cells (MSC). XF MSC-OEC spheroids were characterized in detail and compared to spheroids cultured in FBS-supplemented medium. XF spheroids presented comparable integrity, size and morphology as the reference culture. The use of both media resulted in spheroids with similar structure, abundant extracellular matrix deposition and specific patterns of OEC distribution and organization. Notably, XF spheroids presented significantly enhanced angiogenic potential, both *in vitro* (fibrin sprouting assay) and *in vivo* (CAM assay). These findings are particularly promising in the context of potential therapeutic applications.

## Introduction

Mesenchymal stem cells (MSC) hold great therapeutic potential due to their multilineage differentiation capacity, distinctive immunosuppressive properties and high expansion potential^1^. Transplanted MSC home to damaged tissues and inflammation sites, constituting a particularly promising population for regenerative medicine applications^2^. MSC play a dual role at injured sites, repopulating damaged tissues by differentiating into specific cell types, as well as promoting the survival and proliferation of host cells through paracrine activity^3^. One common problem faced in CT is the limited cell survival within the host environment^4,5^. It has been shown that the pre-assembling of cells into multicellular aggregates can have a positive impact on their viability and therapeutic action^6,7^. Spheroid culture is a promising approach, promoting the generation of dynamic 3D environments that preserve cell-cell and cell-matrix interactions^4^. MSC spheroids were described to display enhanced anti-inflammatory and angiogenic activities, augmented differentiation and stemness potential, improved survival and delayed replicative senescence^8,9^. With the outbreak of methods for their high-throughput generation and facilitated analysis^10^, spheroids became appealing tools for clinical applications^11^. Different approaches to improve their performance have been suggested, such as co-cultures of different cell types, *in vitro* pre-conditioning and combination with biomaterials^4^. Incorporation of endothelial cells in such constructs was reported to improve their viability and facilitate integration with host vasculature^5^. OEC are particularly attractive for their capacity to structurally contribute to neovessel development^12^.

Yet, broader translation to the clinics is still elusive, as many conceptual, as well as technical issues, arise upon such transition^13^. All too often, animal-derived materials are used for cell expansion, commonly also in the area of cell therapies, as Federal Drug Administration (FDA) reported that in more than 80% of the investigational new drug applications for MSC, FBS was used in the manufacture process^14^. Apart from safety and ethical concerns, the use of FBS is also becoming problematic from economic point of view, as some experts anticipate its critical shortage in the near future^15,16^. Therefore, different alternatives to FBS-supplemented cell culture are being developed, including serum-free and XF systems^16^. Several options for FBS-free expansion of MSC exist, including commercially available replacements^17^. A protocol for the generation of MSC spheroids under defined XF conditions, together with their *in vivo* administration, has been proposed very recently by Ylostalo *et al*.^18^. Regarding endothelial progenitor cell culture, some attempts at establishing FBS-free systems were described, yet no consensus or ‘golden standard’ protocols exist^19–22^. Importantly, no complete XF medium for endothelial cells propagation is currently available on the market.

SCC is an industrial, pharmaceutical-grade human plasma-derived supplement for cell culture, currently under development at Grifols (Barcelona, Spain), manufactured under GMP guidelines^23^. As opposed to most of the reported time-consuming and prone to variations protocols for XF culture of EC, SCC is a ready-to-use lyophilized product. One of its main advantages is the consistency from batch to batch, assured by pooling plasma of at least 1000 donors. The supplement is characterized by high safety, resulting from rigorous testing of donations, as well as specific viral inactivation steps during production, in addition to purification steps with pathogen removal capacity. SCC was already described as a suitable candidate for XF culture of MSC, ESC and iPSC^23–26^.

Here, we present the development of a SCC-based XF formulation for the culture of umbilical cord blood-derived OEC. Following extensive screening of different formulations, OEC growth, phenotype and functional behaviour in the established XF medium was evaluated. The ultimate goal of present work was the generation of 3D MSC-OEC spheroids under XF conditions, and the assessment of their angiogenic potential.

## Results

### Optimization of a XF SCC-based medium for OEC culture

**Figure 1:**
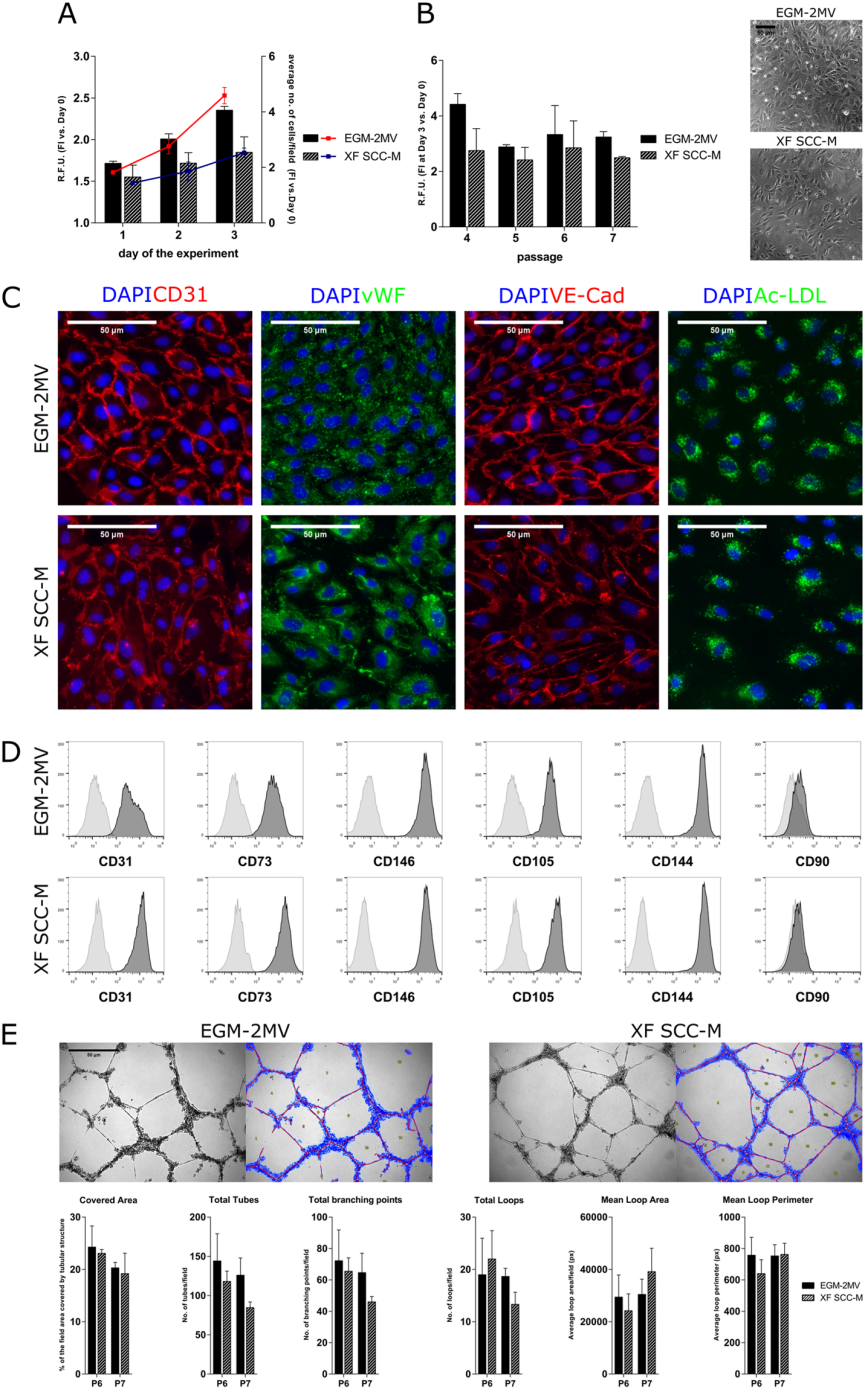
Optimization of the XF SCC-M in OEC culture. (**A**) OEC proliferation in the XF SCC-M. Cell growth of OEC in the control and XF conditions, expressed respectively in the metabolic activity levels and the rate of culture expansion measured by high throughput cell number quantification. (**B**) XF SCC-M formulation performance in terms of cell metabolic activity, analysed over the passages of OEC culture. Phase contrast images of OEC morphology in the control and XF formulation. (**C**) Representative images showing cellular localization of CD31, vWF, VE-Cad, and Ac-LDL in standardly and SCC-grown OEC. (**D**) Representative flow cytometry histograms of OEC cultured in the control medium and XF SCC-M showing reactivity with EC-characteristic marker molecules (right-shifted filled black curves compared with grey lined curves of the appropriate controls) and lack of reactivity with the negative CD90 marker. (**E**) Representative micrographs of the tubular-like structures formed in Matrigel, along with exemplary analyses of OEC grown in EGM-2MV and XF SCC-M. Quantification of characteristic parameters of the tubular structure derived from EGM-2MV and XF SCC-M-cultured cells.

To optimize a SCC-supplemented XF medium for OEC culture, we started by an initial screening phase to test different formulations with varying SCC concentration and addition of different components (SI, Table 1). The resazurin assay was used to estimate cell proliferation levels, as it provides relatively robust data in a time-efficient manner. OEC were routinely grown in the ‘golden standard’ commercially available EGM-2MV (Lonza), which served as the reference medium. Upon screening, the XF medium consisting of serum free cocktail (SFC) with 20% SCC and 10 ng/ml VEGF (from now on designated as XF SCC-based medium or XF SCC-M) showed the best performance and was chosen for further characterization of OEC growth and phenotypic stability. OEC proliferation in XF SCC-M was also evaluated by high-throughput cell number quantification, correlating to the resazurin assay results (Fig. 1A). OEC expanded in XF SCC-M over the course of 2 passages presented typical cobblestone-like morphology (Fig. 1B). Cells stained positively for CD31, VE-cadherin, vWF and were able to incorporate Ac-LDL. No significant differences in the expression and localization profile were observed as compared to cells cultured in EGM-2MV (Fig. 1C). OEC phenotypic stability along passages, with sequential transition from EGM-2MV (P4) to 50%EGM-2MV/50% XF SCC-M (P5), to 100% XF SCC-M (P6 and P7) was evaluated by flow cytometry (FC). After two passages in 100% XF SCC-M, OEC still expressed high levels (>99%) of endothelial markers CD31, CD146, CD105, CD73 and CD144 (>92%), and very low levels (0.36%) of CD90 (Fig. 1D, SI Table 3). Tubulogenesis of XF-grown OEC was evaluated with a Matrigel-based assay. Similarly to cells in EGM-2MV, OEC cultured in XF SCC-M spontaneously organized into tubular structures along 24 h of culture (Fig. 1E). With Wimtube software a set of parameters was quantified to characterize the formed tubular structures. Results showed no significant differences between OEC expanded in XF SCC-M and EGM-2MV.

### Generation of functional MSC-OEC spheroids under XF conditions

**Figure 2:**
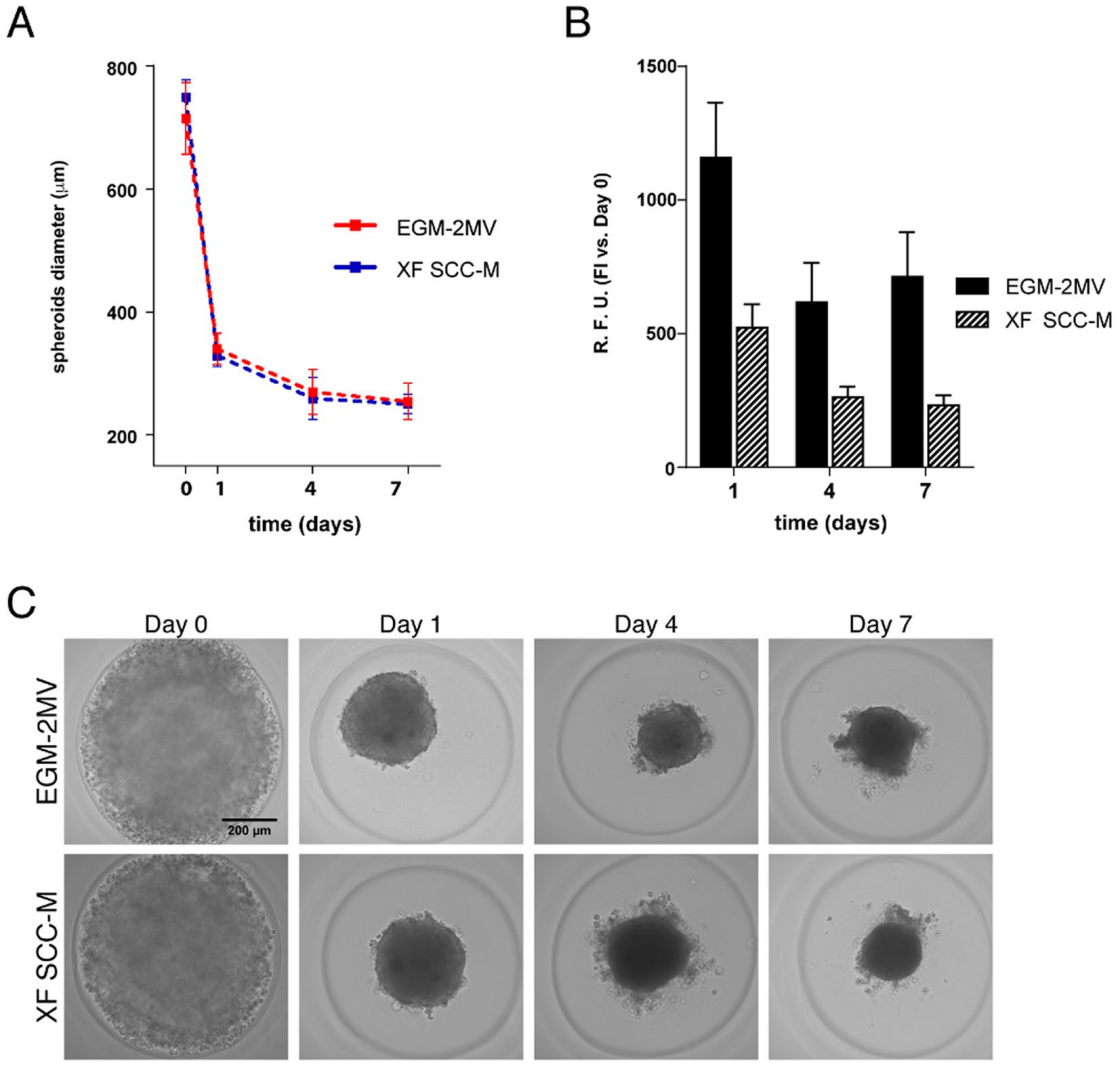
Generation of MSC-OEC spheroids under XF conditions. (**A**) Brigthfield images of MSC-OEC spheroids compaction along time. (**B**) Metabolic activity of the spheroids in culture. (**C**) Morphology of MSC-OEC spheroids over 7-day culture (scale bar 100 μm).

Using microwell array cast technology, it was possible to generate MSC-OEC co-culture spheroids under standard (EGM-2MV) and XF conditions (XF SCC-M). Cells seeded at 1:1 MSC:OEC ratio aggregated within 24 h, forming spherical structures that remained cohesive over 7 days of culture (Fig. 2C). Brigthfield images of spheroids diameter along the time revealed minimal additional compaction (Fig. 2A). No significant differences in terms of diameter size could be observed between spheroids grown under control *vs*. XF conditions. Morphology was also very similar in both conditions, with a group of excluded cells surrounding the spheroid that could be noticed from day 4 of culture (Fig. 2C). A significant decrease in metabolic activity could be observed between days 1 and 4 in both media, followed by nearly constant levels along the remaining period of culture. Despite the values of metabolic activity being lower for spheroids grown in XF SCC-M, the observed trend in terms of fold increase (in relation to day 1) was comparable for both conditions (Fig. 2B).

### ECM production and OEC organization in MSC-OEC spheroids under XF conditions

**Figure 3:**
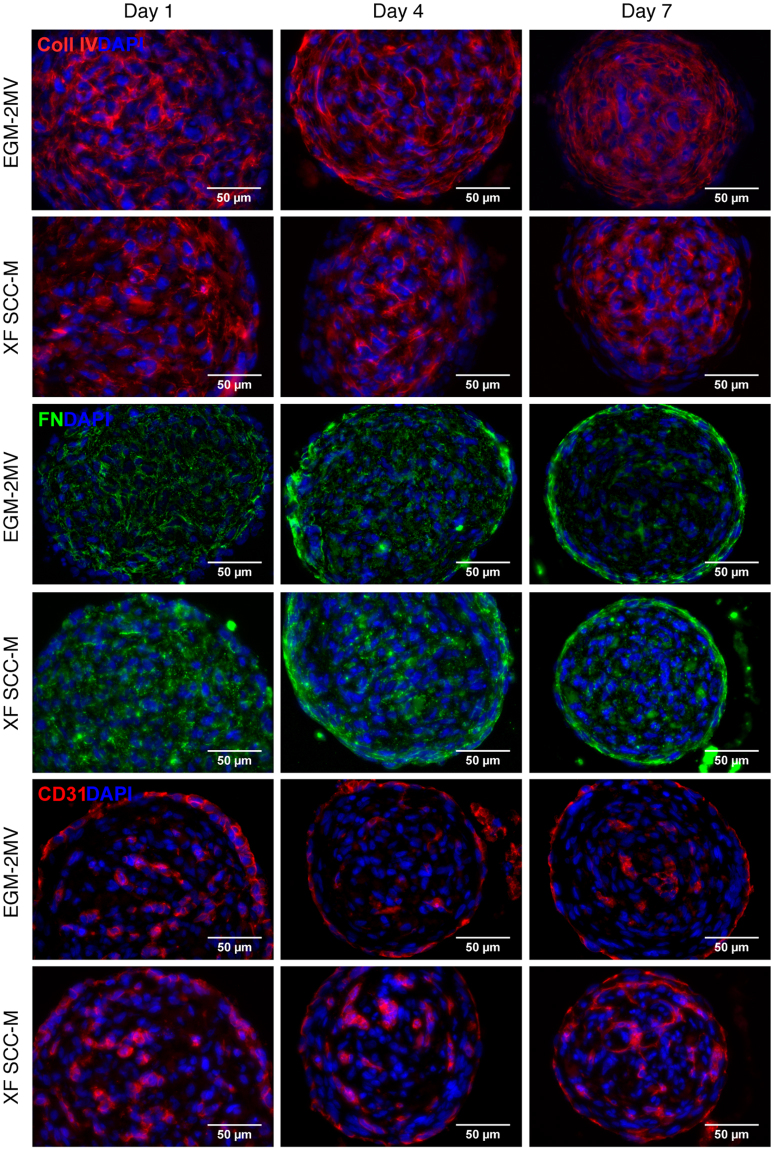
Structure/internal organization of the MSC-OEC spheroids under XF conditions. Representative images showing deposition of ECM proteins (collagen type IV and fibronectin) and cellular organization of the spheroids along the culture, respectively in the control and SCC-based medium immunostaining of collagen type IV (red), FN (green), CD31 (red) counterstained with DAPI (blue). Images were taken with a 40X objective. Scale bar 50 μm.

Extracellular matrix (ECM) production in spheroids is an important event, as it modulates the dynamics of cell-cell and cell-matrix interactions, and cell organization^27^. In order to assess the synthesis of endogenous ECM in MSC-OEC spheroids, immunohistochemical analysis of ECM proteins expression was performed, specifically of fibronectin (FN) and collagen type IV (Col IV). Col IV was found to form extensive meshworks within spheroids, exhibiting similar expression patterns in both conditions (Fig. 3). In EGM-2MV cultured spheroids, fiber assembly into elongated structures was observed in the outer layers, while in XF-grown spheroids a more disorganised mesh was detected. FN expression pattern was very similar in both control and XF culture (Fig. 3). The formation of a layer of polymerized FN fibers at the spheroid periphery was noticed already by day 4, and became more defined along the culture time (Fig. 3). FN deposition at the spheroids core was limited and less organized.

The spatial distribution and organization of OEC, analysed by specific CD31 immunostaining, was similar in the two conditions. OEC partially segregated into the spheroid surface, forming a continuous outer layer, which became more elongated and flattened from day 4 forward (Fig. 3). A gradual transition in the structure of spheroids cores was also observed along the time, with formation of OEC clusters of different size and shape, which were more apparent under XF conditions (Fig. 3). While some of these OEC clusters began to sprout, this did not lead to extensive network formation and cells eventually became apoptotic (data not shown).

### Ultrastructural analysis of MSC-OEC spheroids by transmission electron microscopy (TEM)

**Figure 4:**
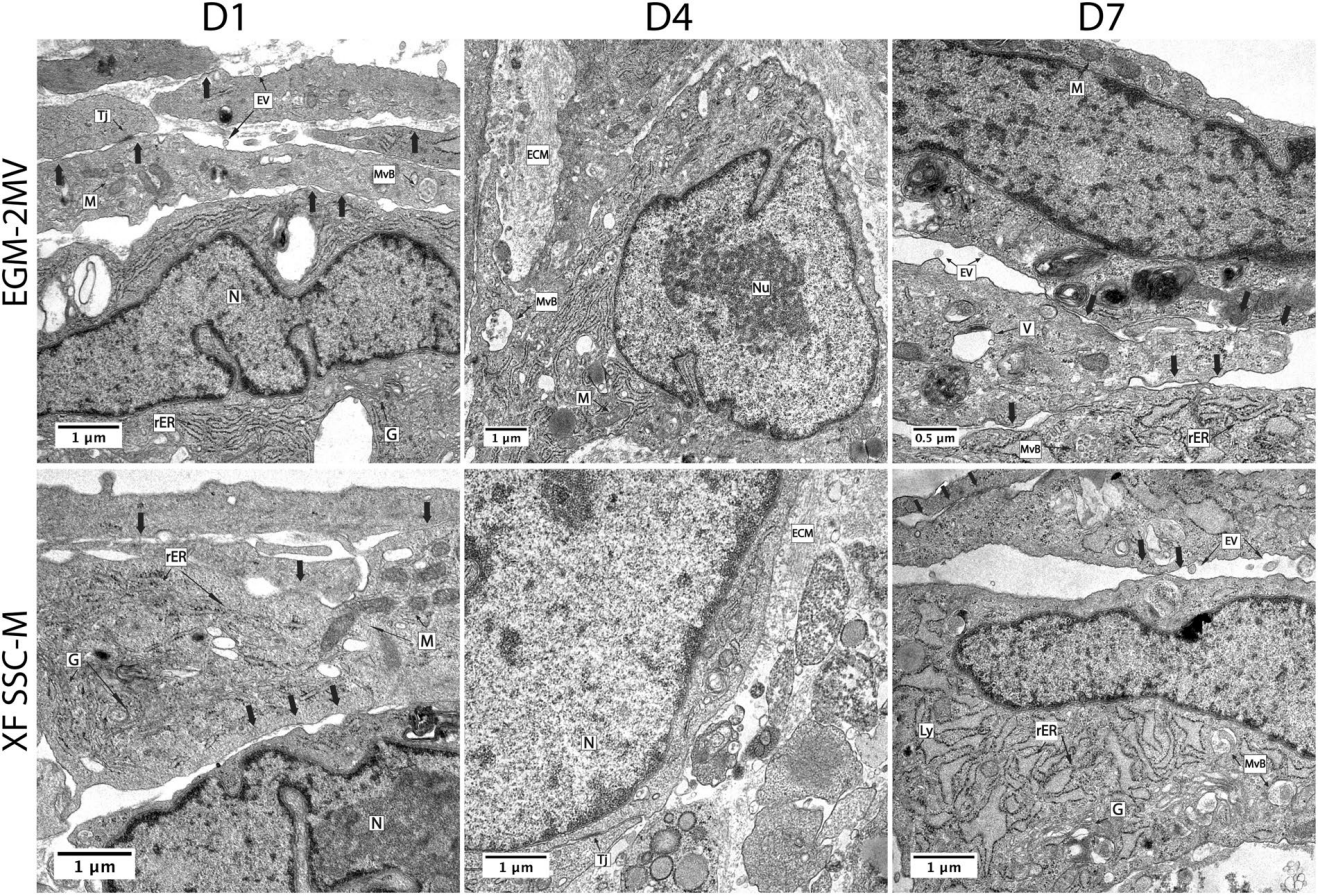
Transmission electron microscopy (TEM) analysis of MSC-OEC spheroids external layers ultrastructure. Images depict elongated cells (arrows); accumulation of ECM – extracellular matrix components Tj – tight junctions and different organelles including N – nuclei; Nu – nucleolus; M – mitochondria; G – golgi apparatus; ER – endoplasmic reticulum; rER – rough endoplasmic reticulum; Ly- lysosomes; V- vacuoles; MvB- multivesicular bodies and EV- extracellular vesicles. Images were obtained with 12 K and 15 K and 35k magnification.

The ultrastructure of MSC-OEC spheroids was analysed by TEM (Fig. 4). Spheroids from both conditions displayed similar features, with elongated cells at the more peripheral layers (arrows) and accumulation of extracellular matrix components, which is in accordance with immunostaining results (Fig. 3). Some tight junctions (Tj) were detected, highlighting cell-cell interactions. Moreover, as depicted in Fig. 4, cells in spheroids exhibited typical organelles. Some microvesicular bodies (MvB) and extracellular vesicles (EV) were also present.

### OEC sprouting from MSC-OEC spheroids under XF conditions

**Figure 5:**
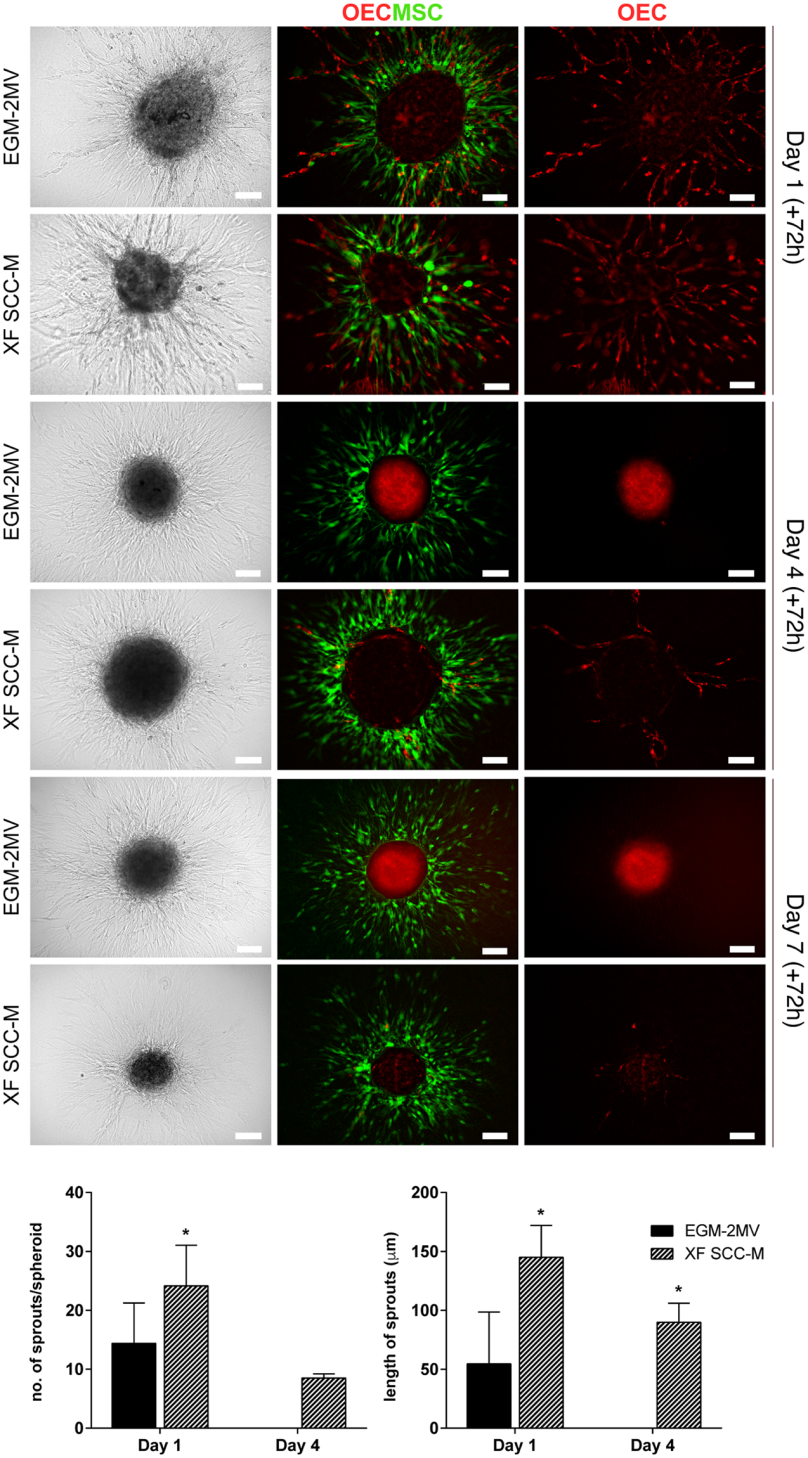
Fibrin-based 3D angiogenesis assay. Representative Brightfield and fluorescence images of sprouting MSC-OEC spheroids, cultured in normal and XF conditions for periods of respectively 1, 4, and 7 days; brought under the assay conditions for 72 h. OEC were marked with DiI-Ac-LDL and MSC were labelled with a CellTracker Green Dye. Scale bars correspond to 100 μm. Graphs represent quantitative analyses of the average numbers of sprouts per spheroid and their length, respectively under normal and XF conditions (n = 7, Mann-Whitney test was used to compare the two groups).

In order to determine their sprouting potential, MSC-OEC spheroids were collected at days 1, 4 and 7 and entrapped in fibrin gel for up to 72 h. For day 1 spheroids, a high degree of outward cell migration into the gel could be observed within 24 h (Fig. 5). There was extensive MSC (labelled with cell tracker green) migration and OEC (labelled in red with Ac-LDL) were shown to extend tubular-like sprouts into the surrounding fibrin matrix, both under control and XF conditions. Yet, with increasing pre-culturing times (day 4 and 7), the level of OEC sprouting in spheroids under XF conditions decreased drastically, and was nearly absent in the controls. Performed quantifications revealed significantly higher average numbers of sprouts per spheroid in XF setting, when compared to control (Fig. 5). The length of OEC sprouting structures assembled in XF SCC-M was also significantly higher (Fig. 5).

### *In vivo* angiogenic potential of MSC-OEC spheroids generated under XF conditions

**Figure 6:**
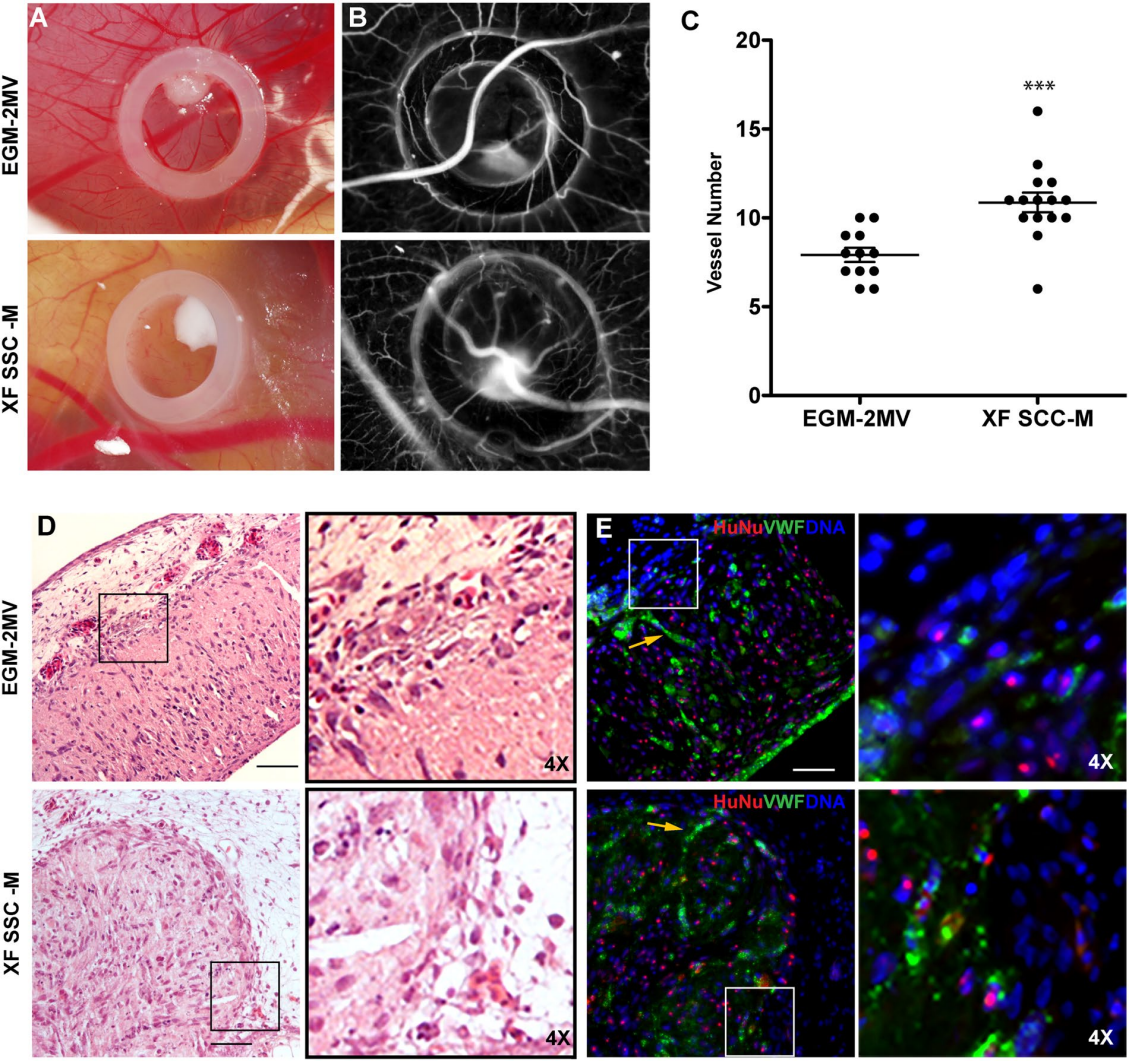
*In vivo* CAM assay. The angiogenic potential of spheroids in both conditions was tested using chick embryonic membrane. (**A**) Brightfield images of spheroids inside the CAM O-ring; (**B**) photomicrographs acquired for counting newly formed vessels; (**C**) Angiogenic effect of spheroids grown in the standard versus XF conditions, expressed by number of newly formed vessels (n ≥ 12, two independent experiments, ***p < 0.0004, Mann-Whitney test was used for statistical comparison). (**D**) HE staining of CAM interface and spheroids after 72 h incubation time (Scale bar 100 μm). (**E**) Immunofluorescence of equivalent sections represented in D (Scale bar 50 μm). vWF (green) was used to access endothelial organization, HuNu (red) to discriminate human nuclei and DAPI (blue) for total nuclei. In images (**D**) and (**E**), insets (and respective 4× magnifications, depicted on the right) highlight the direct contact between spheroids and the CAM. Yellow arrows are pointing to organized OEC inside the compacted spheroids.

Based on results from the fibrin sprouting assay, 1 day of pre-culture seemed to be the most promising maturation time for therapeutic applications. Therefore, to evaluate their *in vivo* angiogenic potential, MSC-OEC spheroids were generated and maturated for 24 h under control and XF conditions and then implanted into the CAM. Angiogenic response was quantified after 3 days of implantation as the number of newly formed capillaries in a defined region. Spheroids seeded onto the CAM merged into a large cell mass (Fig. 6A). Quantitative analysis of vascular density in images from day 3 (Fig. 6B) showed a statistically significant higher number of new microvessels in samples of XF-generated spheroids, as compared to the control (Fig. 6C), which correlates with higher angiogenic potential.

Spheroids organized into a macrotissue-like structure, as depicted by HE and immunofluorescent stainings (Fig. 6D,E, respectively). As verified by human-specific staining, human spheroid-derived cells were in direct contact with host chicken cells. Additionally, inside those large cell constructs, endothelial cells (vWF+ cells) partially aligned into capillary-like structures, in both conditions (Fig. 6E and inset).

## Discussion

One major challenge in CT is ensuring functionality of administered cells within the host environment, without premature loss and/or excessive death^4,5^. Several *in vitro* and *in vivo* studies have suggested that pre-assembled cell aggregates can be more efficient in promoting therapeutic effects than single-cell suspensions^6,7^. In multicellular aggregates, cell-cell and cell-matrix interactions are preserved, promoting cell survival and improving resistance under unfavourable microenvironments^4^. In addition, cell aggregates have been shown to secrete higher amounts of pro-regenerative and immune-modulatory mediators^8,9^. Microvesicles (MV) are likely to play a role in the therapeutic potential of MSC spheroids. While MV derived from MSC spheroids are still poorly explored, a recent study by Xie *et al*. suggests that these may possess enhanced capability of promoting signal factors secretion, as well as enhanced immunomodulatory activities, as compared to MV from MSC monolayer culture^28^. Interestingly, in the present study, microvesicular bodies and extracellular vesicles were detected within MSC-OEC spheroids, suggesting that MV might be involved in cell-cell communication within these 3D systems.

One of the causes of impaired cell survival upon transplantation is the insufficient blood supply leading to shortage of oxygen and nutrients, especially in larger grafts. Addition of endothelial cells of different type and origin to the transplanted multicellular aggregates has been reported to improve the survival of such cell constructs^29,30^. Reports on successful generation of pre-vascularized spheroids for therapeutic strategies can be found in the literature^29,31^, with some studies in animal models showing very promising results^32,33^. However, deeper understanding of the mechanisms of action of such cellular constructs is still needed, especially considering the variety of methodologies and approaches used by different groups. Moreover, the use of animal-origin products, such as FBS, remains one important obstacle in the transition of CT into clinical settings. Here, we optimized a new XF medium for standard monolayer culture of OEC, and then used it for the generation of spheroids for therapeutic application. To the best of our knowledge, this is the first study in which OEC and MSC were combined in self-assembling spheroids, under XF conditions.

Optimization of a XF formulation for OEC culture using SCC, a novel supplement for cell culture, was the first step in the development of our strategy. Human blood-derivatives, used for FBS replacement, were previously shown to support OEC growth^20,21^. Yet, reported methodologies, although efficient in promoting the isolation and propagation of OEC, often depend on complex protocols for the preparation of blood-derivatives, requiring frequent testing of blood donors and approvals from ethical committees. Importantly, substantial variability is always inevitable when applying such approaches. SCC, on the other hand, is an off-the-shelf lyophilized product, convenient for transport and long-term storage and characterized by high consistency ensured by good manufacturing practice. It was previously reported to support the growth of ESC, iPSC and MSC^23–25^. The XF formulation developed here was based on the use of a defined cocktail of GF and supplements described previously in the literature to promote growth of OEC^20,34,35^. The basal medium chosen in the present study was MCDB131, reported to provide particularly favourable conditions for EC proliferation^36^. The optimization process was initiated with 5% SCC supplementation in the place of FBS, to achieve direct comparison with the golden standard EGM-2MV medium. Yet, a dose-dependent improvement in cell proliferation was observed, and SCC concentration was changed to 20%. As such formulation was still not providing sufficient support of OEC growth, additional GF supplementation was necessary. VEGF is known as one of the most potent mitogens for EC^37^; and indeed it proved to be most efficient in enhancing OEC metabolic activity and proliferation. Importantly, cells grown in XF SCC-M maintained typical endothelial morphology, expression of characteristic phenotypic markers and *in vitro* tubulogenic ability, which are key features in the context of potential clinical applications.

Next, the optimized XF formulation was used for generating pre-vascularized MSC-OEC spheroids. With the use of micro-mold arrays, we were able to obtain high numbers of uniformly-sized spheroids in each culture assay. The size of pre-vascularized spheroids can be crucial for their angiogenic potential^4^. In our study, after initial compaction, diameters of generated spheroids were around 250 µm. In excessively large constructs, limited diffusion of oxygen, nutrients and metabolites may compromise cell survival, resulting in the formation of necrotic cores^5^. On the other hand, spheroids larger than 100 µm were reported to exhibit increased VEGF secretion, as compared to the smaller ones, so the selection of spheroids size should be a well-thought-out process^38^. MSC-OEC spheroids, generated both under control and XF conditions, were characterized by a decrease in metabolic activity at the onset of culture. This can possibly be explained by the sudden transition of cells from monolayer (2D) into spheroid culture (3D), as also observed by others^8,39^; and the adaptation of MSC fraction to the medium optimized for OEC culture. We decided to maintain MSC-OEC spheroids in the endothelial culture media, as we aimed at preserving most favourable conditions for OEC, the more sensitive cells in the system. While total levels of metabolic activity were lower in XF culture, the general trend in terms of fold increase along the culture was comparable to EGM-2MV, similarly to what was previously observed in 2D OEC cultures.

The ECM is essential in native tissues, providing not only structural support and cell adhesion sites, but also biochemical and biomechanical signals that regulate cell growth, migration, differentiation and survival^40^. In spheroids, the presence of ECM is important for their structural integrity, and for the establishment of a matrix-dependent communication network. We observed abundant deposition of ECM proteins, namely Col IV and FN, within spheroids generated both in control and XF conditions. The accumulation of ECM components was confirmed by TEM analysis. The presence of such proteins is of particular importance in the context of neovessel formation and stabilization. FN, together with its receptors, is directly involved in vascular morphogenesis^41^. Col IV is essential for the stability and internal organisation of the vascular basement membrane^42^. Moreover, in a clinical application scenario, the presence of endogenous ECM in spheroids is expected to provide a protective environment against aggressive conditions of the host milieu, such as hypoxia and oxidative stress^43^. In particular, it might prevent anoikis, a form of programmed cell death occurring when anchorage-dependent cells lose contact with ECM, a major reason for the limited viability of transplanted single-cells^44^.

Various types of vascular organization in co-culture spheroids have been reported, with no clear consensus and notable inconsistencies in the interpretation of the – often only qualitative – data^29,31,45–47^. Here, by immunostaining, we observed the formation of a concentric layer of OEC at the spheroids periphery, both under normal and XF conditions. The presence of elongated cells at the more peripheral layers was also confirmed by TEM^45^. In a study by Hsu *et al*., the same kind of cell patterning was described to have particularly high angiogenic potential^46^. In most reports on pre-vascularized spheroids, populations of adult EC, such as HUVEC, were used. Here, we have selected OEC, as they constitute an easily available population of high proliferative cells with vasculogenic potential, with higher clinical relevance. In a comparative study by Rouwkema *et al*., different types of EC were examined in terms of their ability to form primitive vascular structures within co-culture spheroids, and OEC were indeed reported to organize into most advanced vascular networks^12^. In the current study, we observed the formation of OEC clusters with sprouting cells within the cores of MSC-OEC spheroids, more apparent under XF conditions, but these eventually became apoptotic. In co-culture spheroids, MSC can act as structural support for the microvascular formations, by producing ECM and acting as mural cells, as well as promoting angiogenesis and cell viability through paracrine mechanisms^5,48^. A number of groups reported increased ratios of the supporting cells in pre-vascularized spheroids to have positive impact on their overall vasculogenic function^47,49,50^. Therefore, it could be valuable to test different cell seeding numbers and ratios in our system, and verify if these could eventually stabilize the sprouting OEC clusters.

Our observations of aligned OEC in the outer layers of spheroids led us to extend evaluation of their angiogenic potential in more complex models of *in vitro* fibrin-based assay and *in vivo* CAM assay. Quantitative analyses revealed that spheroids grown in XF SCC-M resulted in significantly higher sprouting in fibrin gel and in higher numbers of neovessels in CAM. In another report, similar tendency of enhanced angiogenic potential was observed when FBS was substituted by human serum in a multilayer co-culture system^47^. In fibrin, a significant decrease of OEC sprouting was observed with extended culture times in both settings, more obvious in EGM-2MV. We observed a fraction of excluded cells surrounding spheroids, appearing by day 4 of the culture, which we assumed to be OEC as they were CD31+ (data not shown). Therefore, potentially reduced numbers of OEC in the spheroids could in fact reflect the lower endothelial sprouting observed at later time points. Notably, a significant loss of endothelial cells in pre-vascularized spheroids throughout the culture was reported before^49^. The higher angiogenic potential of XF-grown spheroids could be potentially attributed to relatively high amounts of VEGF present in the XF SCC-M. VEGF is a well-established modulator of angiogenesis in both normal and pathological conditions^51^. It has to be noted however, that concentrations of growth factors in the EGM-2MV medium are not disclosed by the manufacturer, nor is defined the composition of FBS and SCC, so any definite conclusions about the mechanisms of action of both media are not feasible.

Spheroids were described by some groups as building blocks in bottom-up methods for the formation of larger structures, usually referred to as macrotissues^4^. That approach has already shown some promising results in strategies aiming at regeneration of bone^52^, skin^53^ and hepatic tissue^54^. Interestingly, upon CAM implantation, spheroids effectively fused into larger and somewhat organized structures. Some elongated OEC arrangements were detected within these macrotissues.

Summing up, formation of 3D cellular spheroids under FBS-free conditions was reported before, but mostly in applications other than therapeutic vascularization^39,47,55^. Here, we show that XF spheroids present no differences in terms of morphology, size, ECM deposition and OEC organization, as compared to FBS-grown spheroids, while exhibiting enhanced angiogenic potential. Thus, overall, our results suggest that the XF SCC-M formulation may in fact be more suitable for clinical applications than FBS-supplemented media. That is a particularly promising finding, as not only we were able to obtain an animal component-free setting that is crucial for clinical applications, but that also improves the proangiogenic performance of MSC-OEC spheroids.

## Conclusions

This study describes a novel strategy for the development of XF pre-vascularized spheroids with enhanced angiogenic potential. We demonstrated that MSC-OEC microtissues cultured in the presence of SCC were able to maintain their phenotype and function. We further proved that the XF formulation supported the formation of vascular-like structures more efficiently than the standard medium, both *in vitro* and *in vivo*. Presented work contributes to the advancement of FBS-free cell culture systems, which we strongly believe to be necessary for the application of any cell-based therapeutic strategy in tissue engineering and regenerative medicine.

## Materials and Methods

### Cells and media

OEC were isolated from human umbilical cord blood of healthy donors, according to protocols approved by the UC Davis Stem Cell Research Oversight Committee, as described previously^56^, and were a kind gift from Eduardo Silva (UC Davis UCB). All blood donors were kept anonymous, so the need for written consent was waived. Cells between passages 4 and 7 were used for experiments. EGM-2MV (Lonza) medium was prepared according to manufacturer’s instructions, by adding provided aliquots (5% FBS, ascorbic acid, hydrocortisone, EGF, VEGF, FGF-β, IGF-1) and 1% Penicillin/Streptomycin (P/S) to the basal medium (EBM-2). Other formulations were prepared on the base of a serum-free cocktail (SFC), consisting of MCDB131 basal medium (BM) supplemented with different growth factors (GF) (Supplementary Information, SI, Table 1), 1 µg/ml ascorbic acid, 0.2 µg/ml hydrocortisone, 2 mM L-glutamine (Biowest) and 1% P/S. All supplements were reconstituted in 0.1% human serum albumin solution in phosphate buffered saline (PBS, pH 7.4) The XF SCC supplement was prepared by reconstitution of the freeze-dried product in MCDB131, followed by filtration (0.2 μm). For optimization of a SCC-based XF medium, SFC was supplemented with 5% FBS (Biowest) or with increasing concentrations of SCC and GF to obtain different in-house formulations (SI, Table 1). Human bone marrow-derived MSC (Poietics, Lonza), between passages 6 and 8, were cultured in DMEM (Thermo Fisher Scientific) supplemented with 10% FBS (Hyclone, GE Healthcare) and 1% P/S. Cells were routinely cultured in T75 flasks at 37 °C under a 5% v/v CO_2_ humidified atmosphere, and passaged upon reaching 70–80% confluence. Culture medium was changed every two days for OEC and twice a week for MSC.

### Optimization of XF media for OEC monolayer culture

#### Cell metabolic activity and proliferation

To assess metabolic activity of OEC grown in different conditions, cells were plated at 10000 cells/cm^2^ in 24-well plates and cultured in respective media. At days: 0 (minimum 4 h upon seeding), 1, 3 and 5 cell metabolic activity was evaluated by Resazurin assay as described before^57^. All assays were performed in triplicates. To evaluate cell proliferation, OEC were seeded in 24-well plates in FBS-supplemented and XF media and grown for 0, 1, 2, 3, 4 and 5 days. They were then fixed with 4% v/v paraformaldehyde (PFA, 15 min), permeabilized in 0.1% Triton X-100 in PBS (15 min) and blocked with 5% FBS in PBS (1 h) at room temperature (RT). Nuclei were counterstained with DAPI (10 min). Samples were washed with PBS and brought under high content analysis. Plates were scanned and images were collected with the IN Cell Analyzer 2000 (GE Healthcare) at 10x magnification with 5000 events/well. The collected data was processed with Spotfire software (TIBCO Software Inc.) and OEC proliferation rate in different conditions was assessed by quantification of the average number of cells per defined field.

#### Immunophenotyping by flow cytometry

Expression profile of typical EC markers (CD31+ , CD73+ , CD144+ , CD146+ , CD105+ and CD90−) along different passages was analysed by flow cytometry (FC). Culture of OEC was initiated in P4 in EGM-2MV. Upon reaching confluence, cells were split and seeded again at 5000 cells/cm^2^ in the formulation consisting of 50% EGM-2MV and 50% XFM (P5). Cells were then cultured for 2 additional passages (P6 and P7) in 100% XFM and EGM-2MV (control). At each passage, cells were harvested, washed with PBS and incubated at 4 °C for 1 h in 50 μl of FC buffer (0.01% sodium azide/0.5% BSA in PBS) with specific concentrations of primary antibodies (SI, Table 2). When necessary, this was followed by a wash with PBS and incubation with secondary antibody (4 °C, 1 h). Samples were analysed using a FACS Calibur (BD Biosciences) and 10000 events per sample were acquired each time. Obtained data was analysed with FlowJo software.

#### Immunofluorescent staining for EC markers

OEC were seeded in 24-well plates in FBS- or SCC-supplemented media and grown for 5 days. To evaluate their ability to uptake acetylated low-density lipoprotein (Ac-LDL), OEC were incubated (4 h, 37 °C) with DiI-Ac-LDL labelling agent (Biomedical Technologies Inc.) diluted at 10 µg/ml in media. Cells were fixed with 4% v/v PFA (15 min), permeabilized in 0.1% Triton X-100 in PBS (15 min) and blocked with 5% FBS in PBS (1 h, RT). Subsequently, OEC were incubated with primary anti human antibodies overnight (ON) at 4 °C. Cells were washed 3X with 0.05% Tween 20 and incubated with secondary antibodies (1 h, RT) (SI, Table 2). Nuclei were counterstained with DAPI (10 min), samples were washed with PBS and imaged at 20x magnification with inverted fluorescence microscope (Axiovert M100. Carl Zeiss).

#### Matrigel sprouting assay

For the Matrigel assay, OEC were expanded in control and XF medium as described. Upon subculture at P6 and P7, 6.25 × 10^4^ cells/cm^2^ were plated on Matrigel-coated 96-well plates and incubated at 37 °C in 5% CO_2_ for 24 h. Brightfield images of tubular structures were acquired with an inverted microscope (Axiovert M100, Carl Zeiss) using 5x objective and further analysed with Wimtube software (Wimasis, Córdoba, Spain).

### Generation of MSC-OEC spheroids under XF conditions

Spheroids were produced using commercially available MICROTISSUES® technology (MicroTissues Inc.). Briefly, 12-series agarose micro-molds were prepared using 2% agarose in 0.9% of NaCl. Micro-molds were placed in 12-well plates and equilibrated with either EGM-2MV or XF SCC-M, before cell seeding. OEC and MSC were resuspended in each media and seeded in 1:1 ratio at a concentration of 3.24 × 10^5^ cells/micro-mold, corresponding approximately to 4000 cells per spheroid. A period of 30 min incubation was used for cells to settle into the micro-molds and additional medium was finally added to the wells. Cells were grown in parallel in both media, along 7 days, with media changes every 48 h. Cultures were monitored with Brightfield microscopy (ZOE™ Fluorescent Cell Imager, Bio-Rad Laboratories).

#### Cell metabolic activity in MSC-OEC spheroids

Metabolic activity of spheroid cultures was assessed using Resazurin assay as described in the previous section. At day 1, 4 and 7, spheroids (≥10/condition) were collected from micro-molds and placed in 96-well plate for the assay, always performed in triplicate. Obtained fluorescence values were normalized in relation to number of spheroids placed in each well.

#### Immunohistochemical analysis of MSC-OEC spheroids

Spheroids were fixed at days 1, 4 and 7, directly in the agarose molds, with 4% w/v PFA (30 min, RT). Fixed samples were paraffin-embedded and sectioned into 6 µm sections. For staining, sections were deparaffinized in xylene and rehydrated in sequentially decreasing ethanol concentrations. Antigen retrieval was performed with TE (10 mM Tris-1 mM EDTA, pH 9) or sodium citrate (10 mM, pH 6), at 96 °C for 30 min. Sections were permeabilized in 0.25% Triton X-100 and blocked with 5% FBS in PBS (1 h, RT). Subsequently, sections were incubated with primary anti human antibodies (ON, 4 °C) (SI, Table 2). Sections were then washed with PBS and incubated with secondary antibodies (1 h, RT). Nuclei were counterstained with DAPI (10 min) and samples were mounted with VectaShield (H-1000, Vector). Sections were imaged with Zeiss AxioImager Z1 microscope (Carl Zeiss, Germany) equipped with an AxioCam MR ver.3.0.

#### Analysis of MSC-OEC spheroids by transmission electron microscopy (TEM)

Spheroids were retrieved from the agarose molds at days 1, 4 and 7, washed in PBS and fixed ON in 2 wt% glutaraldehyde/2.5 wt% paraformaldehyde solution in 0.1 M sodium cacodylate (pH 7.4). The spheroids were stained ON at 4 °C, in a 2%(v/v) osmium tetraoxide solution in 0.1 M sodium cacodylate; followed by 3 washes in 0.1 M cacodylate for 10 min each. To enhance the membrane staining, spheroids were transferred to 1% (v/v) uranyl acetate for 1 h at 4 °C in the dark. Spheroids were then immobilized in HistogelTM and dehydrated in a gradient series of ethanol solutions: 70%, 80%, 90%, 100% (v/v) and finally in a propylene oxide solution for 10 min each. Inclusion in EPON resin was performed by immersion of spheroids in a gradually increasing series of propylene oxide to EPON as follows: 3:1, 1:1, 1:3 and 0:1 for 1 h each. At the end, inclusion of spheroids in EPON resin was performed in a silicon mold. EPON polymerization took place at 60 °C for 48 h. Sections with 60 nm thickness were prepared using a diamond knife (Diatome, Hatfield, PA, USA) and were recovered to 200 mesh Cu-grids. Staining of sections using 2 wt% uranyl acetate and saturated lead citrate solution was performed before observation. Visualization was performed at 80 kV in a Jeol JEM-1400 transmission electron microscope (Japan) and digital images were acquired using a CCD digital camera Orious 1100 W (Tokyo, Japan).

#### Fibrin sprouting assay

Prior to seeding in agarose micro-molds, MSC were labelled with CellTracker green fluorescent dye (ThermoFisher Scientific). Culture under standard and XF conditions was performed as described and spheroids were collected for the assay at days 1, 4 and 7. To prepare fibrin gels, 4 mg/ml fibrinogen solution in 0.9% NaCl was prepared, mixed with aprotinin and spheroid suspension and added to each well of a 24-well plate. Thrombin (2 U/ml in PBS) was added immediately and the whole solution was well mixed and left to polymerize (30 min, 37 °C). Fibrin gels were then cultured in each medium with daily media changes. After 72 h, fibrin-embedded spheroids were incubated with DiI-Ac-LDL (10 µg/ml) and images were acquired with ZOE™ Fluorescent Cell Imager (Bio-Rad Laboratories). Numbers and length of sprouts per spheroid were quantified in randomly selected spheroids from each condition.

#### CAM assay

*In vivo* chick chorioallantoic membrane (CAM) assay was performed using fertilized White Leghorn eggs incubated at 37.8 °C and 60% humidity. At embryonic day 3 (E3), 1.5–2 mL of albumin was removed to allow the detachment of the developing CAM and a square window was opened in the shell. MSC-OEC spheroids were generated as described, collected from micro-molds at day 1, washed with PBS and placed directly on top of CAM at E10, within a confining O-ring. Eggs were re-sealed and returned to the incubator, being hydrated every day. After 3 days, cover tapes were removed and embryos were euthanized by adding a fixative. Samples of CAM tissue in the O-ring region were excised and examined under a stereomicroscope to quantify the numbers of newly formed vessels (<20 µm diameter) growing radially towards the ring area. Quantifications were performed in a blind fashion manner. Excised tissues were paraffin-embedded and cut (3 μm or 6 μm sections) for histological and immunohistochemical analysis. Samples were stained with Hematoxylin/Eosin (HE) to assess morphological features and imaged using a Zeiss Axioskop 2 microscope. Human cell location and OEC organization were assessed by immunofluorescence using specific markers (SI, Table 2). Z-series optical sections were acquired using a Zeiss AxioImager Z1 (Carl Zeiss, Germany) equipped with an AxioCam MR ver.3.0. All experiments were carried out according to European Directive 2010/63/EU and the national Decreto-Lei n°113/2013.

### Statistical analysis

Results are shown as the mean ± standard deviation (SD). For groups of data that followed the parametric distribution two-way ANOVA was used, for non-parametric data Mann-Whitney test was applied. The significant differences were set at p < 0.05. The analyses were performed using GraphPad Prism version 6 software (GraphPad Software Inc., La Jolla, CA).

## Electronic supplementary material

Supplementary information
