# Supplementary material for: Xeno-free pre-vascularized spheroids for therapeutic applications
Source: Sci Rep. 2018 Jan 10;8:230. doi: 10.1038/s41598-017-18431-6 (PMC5762877; doi:10.1038/s41598-017-18431-6)
Supplement: Supplementary file 1 — Supplementary information [file 41598_2017_18431_MOESM1_ESM.pdf]

Supplemental information

Xeno-free pre-vascularized spheroids for therapeutic applications

Bauman E, Feijão T, Carvalho DTO, Granja PL, Barrias CC

Table 1. Heat-map of screening studies to establish an adequate SCC-based XF formulation.

Formulation	Serum (%)		Growth factors (ng/mL)							Coating		FI	N	P
	FBS	SCC	VEGF	EGF	FGF	IGF	GHK	PDGF	ANG	FN	COL			
EGM-2MV **(control)**	5		*	*	*	*						3.3	10	4-6
SFC-5FBS	5		0.5	5	10	20						3.2	10	4-6
BM-5FBS	5											0.9	4	5-6
SFC (noFBS)			0.5	5	10	20						0.5	1	6
SFC-5SCC		5	0.5	5	10	20						1.5	5	4-6
SFC-5SCC+10VEGF		5	10	5	10	20						1.6	3	5,6
SFC-5SCC+10VEGF, FN		5	10	5	10	20				x		1.6	1	6
SFC-5SCC+10VEGF, COL		5	10	5	10	20					x	1.8	2	5,6
SFC-10SCC		10	0.5	5	10	20						1.9	5	4-6
SFC-10SCC+10VEGF		10	10	5	10	20						1.9	3	5,6
SFC-10SCC+10VEGF, FN		10	10	5	10	20				x		2.0	1	6
SFC-10SCC+10VEGF, COL		10	10	5	10	20					x	2.0	2	5,6
SFC-20SCC		20	0.5	5	10	20						2.2	6	4-6
SFC-20SCC+10VEGF **(XF SCC-M)**		20	10	5	10	20						2.7	10	4-6
SFC-20SCC+10VEGF, FN		20	10	5	10	20				x		2.2	2	4-6
SFC-20SCC+10VEGF, COL		20	10	5	10	20					x	2.8	5	5-6
SFC-20SCC+20VEGF		20	20	5	10	20						4.1	1	6
SFC-20SCC+EGF		20	0.5	10	10	20						2.1	1	5
SFC-20SCC+FGF		20	0.5	5	20	20						2.0	1	5
SFC-20SCC+IGF		20	0.5	5	10	30						2.2	1	5
SFC-20SCC+GHK		20	0.5	5	10	20	50					2.1	1	5
SFC-20SCC+ANG		20	0.5	5	10	20			50			3.5	1	6
SFC-20SCC+10VEGF+EGF		20	10	10	10	20						2.2	1	5
SFC-20SCC+10VEGF+FGF		20	10	5	10	20						2.2	1	5
SFC-20SCC+10VEGF+IGF		20	10	5	10	30						2.1	1	5
SFC-20SCC+10VEGF+GHK		20	10	5	10	20	50					2.4	2	4,5
SFC-20SCC+10VEGF+GHK. FN		20	10	5	10	20	50			x		2.3	1	4
SFC-20SCC+10VEGF+50ANG		20	10	5	10	20			50			3.9	1	6
SFC-20SCC+20VEGF+50ANG		20	20	5	10	20			50			3.9	1	6
SFC-20SCC+FGF+EGF		20	0.5	10	20	20						2.1	1	4
SFC-20SCC+FGF+EGF. FN		20	0.5	10	20	20				x		1.8	1	4
SFC-20SCC+10VEGF+EGF+GHK		20	10	10	10	20	50					2.0	1	5
SFC-20SCC+10VEGF+FGF+GHK		20	10	5	20	20	50					2.0	1	5
SFC-20SCC+10VEGF+GHK+PDGF		20	10	5	10	20	50	20				1.9	2	4,5
SFC-20SCC+10VEGF+GHK+PDGF, FN		20	10	5	10	20	50	20		x		2.1	1	4
SFC-20SCC+10VEGF+EGF+FGF+GHK		20	10	10	20	20	50					2.1	1	5
EndoGoXF (+Supplement Mix)			**	**	**	**	**	**	**			1.7	1	6
EndoGoXF+20SCC		20	**	**	**	**	**	**	**			4.0	1	6
EndoGoXF+20SCC+10VEGF		20	10**									4.0	1	6
EndoGoXF+20SCC+10VEGF, COL		20	10								x	3.8	1	6

COLOUR CODE

FI (Fold Increase D3/D0):	0.5 to 1.4	1.5 to 2.4	2.5 to 3.4	3.5 to 4.4

Abbreviations: ANG – angiopoietin 1; BM – basal medium; COL – human collagen type I; D0, D3 – Day 0,3 of the culture; FI – fold increase; FN – human fibronectin, GHK – liver cell growth factor; N – number of individual experiments; PDGF – platelet-derived growth factor BB; SFC – serum-free cocktail. Notes: 1) (*), (**) – concentrations not disclosed by the manufacturer. 2) EGM-2MV and EndoGoXF are designations of commercial media (from Lonza and Biological Industries, respectively). 3) In some cases (formulations with high VEGF concentration (20 ng/mL) and those containing ANG, depicted values are higher than those of the reference medium. Yet, in the individual experiments, those formulations did not yield FI values greater than EGM-2MV nor the optimized XFM, and for that reason they were not selected for further analysis.

Table 2. Antibodies used in respective immunofluorescence-based assays.

Antibody	Species source	Manufacturer	Antibody dilution
**Immunofluorescent staining of OEC for EC-characteristic markers**			
anti CD31	mouse	Dako	1:100
anti VE-Cad/CD144	mouse	Santa Cruz	1:100
anti von Willebrand factor	rabbit	Dako	1:200
anti-mouse Alexa Fluor 594	goat	ThermoFisher Scientific	1:1500
anti-rabbit Alexa Fluor 488	goat	ThermoFisher Scientific	1:1500
**Immunohistochemical analysis of MSC-OEC spheroids**			
anti CD31	mouse	Dako	1:150
anti Fibronectin	rabbit	Sigma	1:200
anti Collagen type IV	mouse	Dako	1:100
anti-mouse Alexa Fluor 594	goat	ThermoFisher Scientific	1:1000
anti-rabbit Alexa Fluor 488	goat	ThermoFisher Scientific	1:1000
**Immunohistochemical analysis of excissed CAM tissue**			
anti von Willebrand factor	rabbit	Dako	1:300
anti Human Nuclear Antigen	mouse	Millipore	1:400
anti-mouse Alexa Fluor 594	goat	ThermoFisher Scientific	1:1000
anti-rabbit Alexa Fluor 488	goat	ThermoFisher Scientific	1:1000

Table 3. Flow cytometry analysis of OEC sequentially transferred from EGM-2MV to XF SCC-based medium.

**Cell Passage**	**Medium**	**Endothelial markers***					**Negative marker***
		**CD31**	**CD73**	**CD146**	**CD144**	**CD105**	**CD90**
P4	EGM-2MV	99.17%	99.53%	99.97%	98.75%	92.83%	0.76%
P5	EGM-2MV	99.96%	97.32%	100%	99.96%	99.34%	5.04%
	50%EGM-2MV 50% XF SCC-M	99.92%	99.96%	100%	92.07%	99.4%	1.37%
P6	EGM-2MV	96.10%	98.80%	100%	99.99%	99.46%	1.89%
	XF SCC-M	99.92%	99.94%	99.90%	99.95%	98.94%	1.08%
P7	EGM-2MV	97.31%	97.81%	99.94%	99.94%	98.8%	1.00%
	XF SCC-M	99.54%	99.72%	99.81%	99.97%	99.8%	0.36%

Notes: (*) Percentage of positive cells. The conjugated mouse anti-human primary antibodies used included: anti CD31-fluorescein isothiocyanate (FITC) (BD Biosciences), anti CD73-phycoerythrin (PE) (BD Biosciences), anti CD146-Alexafluor647 (Bio-Rad), anti CD105-FITC (Immunotools) and anti CD90-PE (BD Biosciences). Staining with mouse anti-CD144 primary antibody (Santa Cruz) was followed by incubation with secondary anti-mouse Alexafluor647 antibody (ThermoFisher Scientific).
